# Crystal structure, Hirshfeld surface analysis and DFT study of 1-ethyl-3-phenyl-1,2-di­hydro­quinoxalin-2-one

**DOI:** 10.1107/S2056989020015819

**Published:** 2021-01-01

**Authors:** Gamal Al Ati, Karim Chkirate, Ashraf Mashrai, Joel T. Mague, Youssef Ramli, Redouane Achour, El Mokhtar Essassi

**Affiliations:** a Laboratory of Heterocyclic Organic Chemistry URAC 21, Pharmacochemistry Competence Center, Av. Ibn Battouta, BP 1014, Faculty of Sciences, Mohammed V University, Rabat, Morocco; b Department of Pharmacy, University of Science and Technology, Ibb Branch, Ibb, Yemen; c Department of Chemistry, Tulane University, New Orleans, LA 70118, USA; d Laboratory of Medicinal Chemistry, Drug Sciences Research Center, Faculty of Medicine and Pharmacy, Mohammed V University in Rabat, Morocco

**Keywords:** crystal structure, di­hydro­quinoxaline, hydrogen bond

## Abstract

In the title mol­ecule, C_16_H_14_N_2_O, the di­hydro­quinoxaline moiety is not planar as there is a dihedral angle of 4.51 (5)° between the constituent rings. In the crystal, C—H⋯O hydrogen bonds form helical chains about the crystallographic 2_1_ screw axis in the *b*-axis direction. Hirshfeld surface analysis indicates that the most important contributions to the crystal packing are from H⋯H (51.7%), H⋯C/C⋯H (26%) and H⋯O/O⋯H (8.5%) inter­actions. The optimized structure calculated using density functional theory (DFT) at the B3LYP/6–311 G(d,p) level is compared with the experimentally determined structure in the solid state. The calculated HOMO–LUMO energy gap is 3.8918 eV.

## Abstract 2

The di­hydro­quinoxaline moiety in the title compound is not planar. In the crystal, C—H⋯O hydrogen bonds form helical chains about the crystallographic 2_1_ axes. The chains pack with normal van der Waals contacts.

## Chemical context   

Nitro­gen-based structures have attracted attention in recent years because of their inter­esting properties in structural and inorganic chemistry (Chkirate *et al.*, 2019 ▸; 2020*a* ▸,*b* ▸). The family of nitro­genous drugs, particularly those containing the quinoxaline moiety, is important in medicinal chemistry because of their wide range of pharmacological activities, which include anti­cancer, anti-inflammatory, anti­bacterial, anti­tuberculosis, anti-glycation, anti-analgesic and anti­fungal properties, and for their anti­oxidant potential. In particular, quinoxalin-2-one derivatives are active anti-tumor agents with tyrosine kinase receptor inhibition properties (Galal *et al.*, 2014 ▸). They can also selectively antagonize the glycoprotein in cancer cells (Sun *et al.*, 2009 ▸). Quinoxalin-2-one derivatives are also potential antagonist ligands for imaging the A2A adenosine receptor by positron emission tomography (PET) (Holschbach *et al.*, 2005 ▸). Given the wide range of therapeutic applications for such compounds, we have already reported a route for the preparation of quinoxalin-2-one derivatives using *N*-alkyl­ation reactions carried out with di-halogenated carbon chains (Benzeid *et al.*, 2011 ▸); a similar approach yielded the title compound, C_16_H_14_N_2_O, (I). In addition to the synthesis, we also report the mol­ecular and crystal structure along with a Hirshfeld surface analysis and a density functional theory (DFT) computational study carried out at the B3LYP/6–311 G(d,p) level.
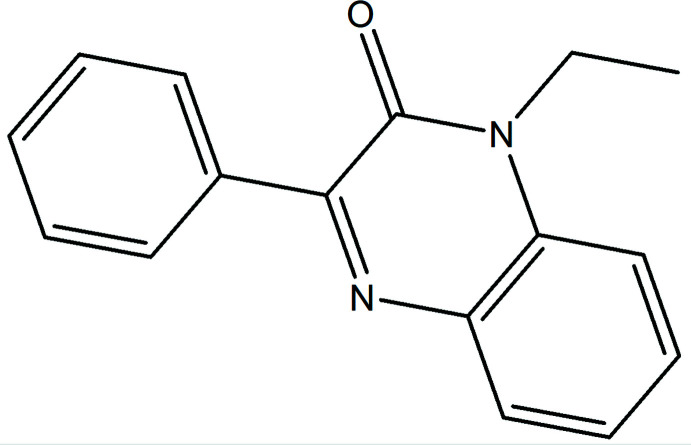


## Structural commentary   

**Figure 1:**
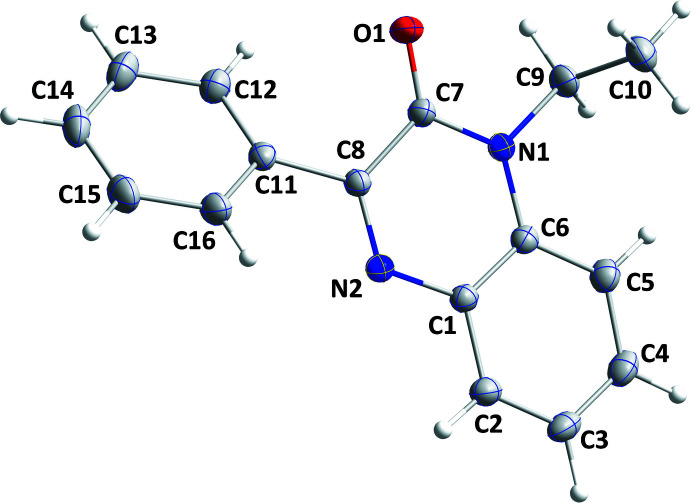
The title mol­ecule with the atom-labelling scheme and 50% probability ellipsoids.

The mol­ecular structure of (I) is depicted in Fig. 1 ▸. The di­hydro­quinoxaline moiety is not planar, as indicated by the dihedral angle of 4.51 (5)° between the constituent rings. Alternatively, the maximum deviations from the mean plane (r.m.s. deviation = 0.060 Å) of the ten-membered, fused ring system are 0.096 (1) Å (C8) and −0.057 (1) Å (C7). The mean planes of the C11–C16 and C1/C6/N1/C7/C8/N2 rings are inclined to one another by 30.87 (4)°. The C6—N1—C9—C10 torsion angle is −78.78 (10)°, indicating the ethyl substituent is rotated well out of the plane of the di­hydro­quinoxaline moiety (Fig. 1 ▸).

## Supra­molecular features   

**Figure 2:**
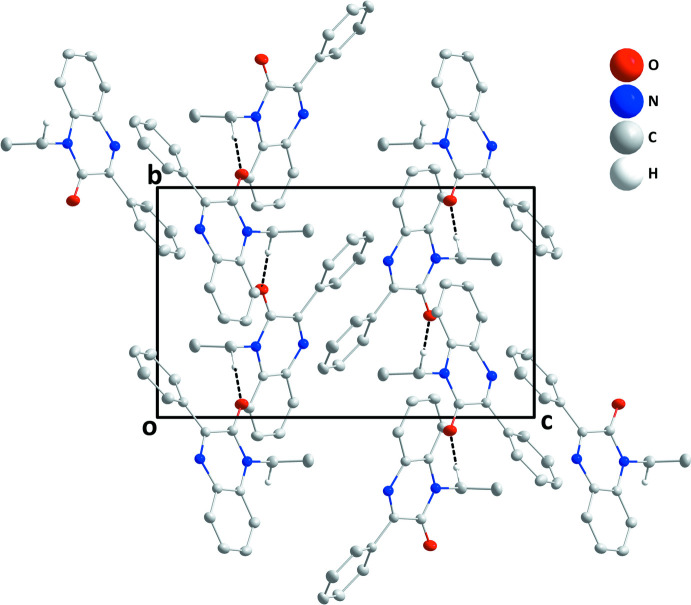
Packing view along the *a*-axis direction with C—H⋯O hydrogen bonds shown as dashed lines.

In the crystal, helical chains about the crystallographic 2_1_ axes are formed by C9—H9*B*⋯O1 hydrogen bonds (Table 1 ▸, Figs. 2 ▸ and 3 ▸). The chains pack *via* normal van der Waals contacts.

## Hirshfeld surface   

**Figure 4:**
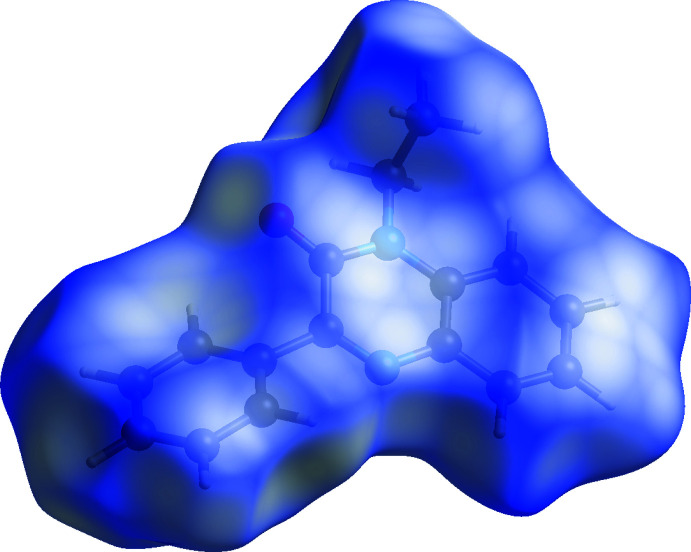
View of the three-dimensional Hirshfeld surface of the title compound, plotted over *d*_norm_.

**Figure 5:**
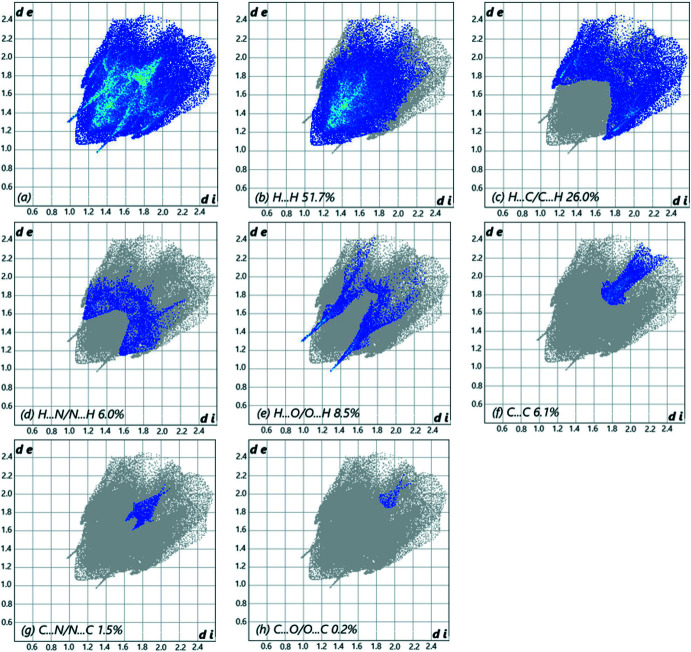
The full two-dimensional fingerprint plots for the title compound, showing (*a*) all inter­actions, and delineated into (*b*) H⋯H, (*c*) H⋯C/C⋯H, (*d*) H⋯N/N⋯H, (*e*) H⋯O/O⋯H, (*f*) C⋯C, (*g*) C⋯N/N⋯C and (*h*) C⋯O/O⋯C inter­actions. The *d*_i_ and *d*_e_ values are the closest inter­nal and external distances (in Å) from points on the Hirshfeld surface.

In order to visualize the inter­molecular inter­actions in the crystal of the title compound, a Hirshfeld surface (HS) analysis (Hirshfeld, 1977 ▸) was carried out using *Crystal Explorer 17.5* (Turner *et al.*, 2017 ▸). A view of the three-dimensional Hirshfeld surface of (I), plotted over *d*_norm_ is shown in Fig. 4 ▸. The overall two-dimensional fingerprint plot (McKinnon *et al.*, 2007 ▸) is shown in Fig. 5 ▸ *a*, while those delineated into H⋯H, H⋯C/C⋯H, H⋯N/N⋯H, H⋯O/O⋯H, C⋯C, C⋯N/N⋯C and C⋯O/O⋯C contacts are illustrated in Fig. 5 ▸ *b*–*h*, respectively, together with their relative contributions to the Hirshfeld surface. The most important inter­actions are H⋯H, contributing 51.7% to the overall crystal packing, which is reflected in Fig. 5 ▸ *b* as widely scattered points of high density due to the large hydrogen content of the mol­ecule, with the tip at *d*_e_ = *d*_i_ = 1.07 Å. For C—H inter­actions, the pair of characteristic wings in the fingerprint plot delineated into H⋯C/C⋯H contacts (26% contribution to the HS), Fig. 5 ▸ *c*, have tips at *d*_e_ + *d*_i_ = 2.79 Å. The pair of scattered points of spikes in the fingerprint plot delineated into H⋯O/O⋯H, Fig. 5 ▸ *e* (8.5%), have the tips at *d*_e_ + *d*_i_ = 2.26 Å. The C⋯C contacts, Fig. 5 ▸ *f* (6.1%), have the tips at *d*_e_ + *d*_i_ = 3.45 Å. The H⋯N/N⋯H contacts, Fig. 5 ▸ *d*, contribute 6% to the HS and appear as a pair of scattered points of spikes with the tips at *d*_e_ + *d*_i_ = 2.67 Å. The C⋯N/N⋯C contacts, Fig. 5 ▸ *g*, contribute 1.5% to the HS, appearing as pair of scattered points of spikes with the tips at *d*_e_ + *d*_i_ = 3.30 Å. Finally, the C⋯O/O⋯C contacts, Fig. 5 ▸ *h*, make only a 0.2% contribution to the HS and have a low-density distribution of points.

## DFT calculations   

**Figure 6:**
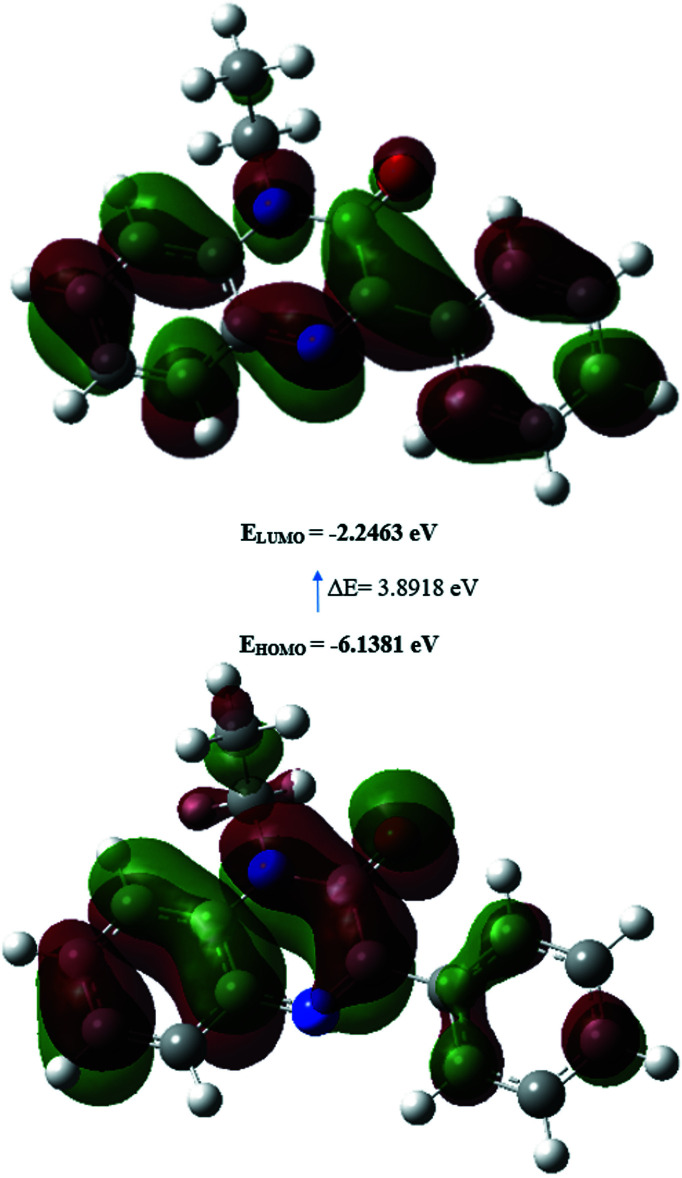
The energy band gap of (I).

The optimized structure of (I) in the gas phase was calculated by density functional theory (DFT) using a standard B3LYP functional and the 6–311 G(d,p) basis-set (Becke, 1993 ▸) as implemented in *GAUSSIAN 09* (Frisch *et al.*, 2009 ▸). The theoretical and experimental results related to bond lengths and angles are in good agreement (Table 2 ▸). Calculated numerical values for (I) including electronegativity (*χ*), hardness (*η*), ionization potential (*I*), dipole moment (*μ*), electron affinity (*A*), electrophilicity (*ω*) and softness (*σ*) are collated in Table 3 ▸. The electron transition from the HOMO to the LUMO energy level is shown in Fig. 6 ▸. The HOMO and LUMO are localized in the plane extending over the whole 1-ethyl-3-phenyl-1,2-di­hydro­quinoxalin-2-one system. The energy band gap [*ΔE* = *E*_LUMO_ - *E*_HOMO_] of the mol­ecule is 3.8918 eV, and the frontier mol­ecular orbital energies, *E*_HOMO_ and *E*_LUMO_, are −6.1381 and −2.2463 eV, respectively.

## Database survey   

**Figure 7:**
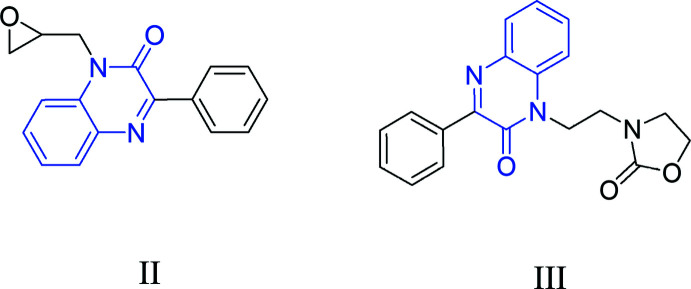
Structures similar to (I): (II) (CSD refcode NIBXEE) and (III) (CSD refcode IDOSUR) obtained in the database search. The search fragment is indicated in blue.

A search of the Cambridge Structural Database (CSD version 5.40, updated March 2020; Groom *et al.*, 2016 ▸) with the quinoxaline-2-one fragment yielded multiple matches. Of these, two had a phenyl at position 3 and are thus most comparable to (I). The first [(II), refcode NIBXEE; Abad *et al.*, 2018*a* ▸)] has (oxiran-2-yl) methyl on nitro­gen 1, and the second [(III), IDOSUR; Daouda *et al.*, 2013 ▸)] has a 3-ethyl­oxazolidin-2-one on nitro­gen 1 (Fig. 7 ▸). Other structures having the quinoxaline-2-one moiety were observed by changing the substituents of positions 1 and 3 in the examples NAYTAJ (1-ethyl; Mamedov *et al.*, 2005*a* ▸), DUSHUV01 (1-benzyl-3-methyl; Ramli *et al.*, 2018 ▸), DUMRUB {1-([1-(3-azido-2-hy­droxy­prop­yl)-1*H*-1,2,3-triazol-4-yl]meth­yl)-3-meth­yl; Abad *et al.*, 2020 ▸}, HIRZOA {1- [(1-butyl-1*H*-1,2,3-triazol-4-yl)meth­yl]-3-methyl; Abad *et al.*, 2018*b* ▸} and SENYUG [3- (indolizin-2-yl)-1-ethyl; Mamedov *et al.*, 2005*b* ▸]. The dihedral angle between the di­hydro­quinoxaline ring system and the phenyl ring is 28.4 (2)° in NIBXEE and the N—C—C— O torsion angle is 87.8 (5)°; the mean plane through the fused-ring system forms a dihedral angle of 30.72 (5)° with the attached phenyl ring. The mol­ecular conformation is enforced by C—H⋯O hydrogen bonds in IDOSUR. In (I), the di­hydro­quinoxaline moiety is not planar, as indicated by the dihedral angle of 4.51 (5)° between the constituent rings. The phenyl ring is tilted towards the pyrazine ring by 30.87 (4)°, which is approximately the same as in IDOSUR but more tilted than in NIBXEE.

## Synthesis and crystallization   

To a solution of 3-phenyl­quinoxalin-2(1*H*)-one (0.7 g, 0.0032 mol) in *N*,*N*-di­methyl­formamide (20 ml) were added bromo­ethane (0.48 ml), potassium carbonate K_2_CO_3_ (0.5g, 0.004 mol) and a catalytic qu­antity of tetra-*n*-butyl­ammonium bromide. The reaction mixture was stirred at room temperature for 24 h. The solution was filtered and the solvent removed under reduced pressure. The residue thus obtained was separated by chromatography on a silica gel column using a hexa­ne/ethyl acetate 9:1 mixture as eluent. The solid obtained was recrystallized from ethanol solution to afford colourless plates of the title compound (yield: 85%).

## Refinement   

Crystal data, data collection and structure refinement details are summarized in Table 4 ▸. Hydrogen atoms were included as riding contributions in idealized positions (C—H = 0.95–0.99 Å) with *U*_iso_(H) = 1.2*U*_eq_(C) or 1.5*U*_eq_(C-meth­yl).

## Supplementary Material

Crystal structure: contains datablock(s) global, I. DOI: 10.1107/S2056989020015819/pk2652sup1.cif

Structure factors: contains datablock(s) I. DOI: 10.1107/S2056989020015819/pk2652Isup3.hkl

Supporting information file. DOI: 10.1107/S2056989020015819/pk2652Isup3.cml

CCDC reference: 2047850

Additional supporting information:  crystallographic information; 3D view; checkCIF report

## Figures and Tables

**Figure 3:**
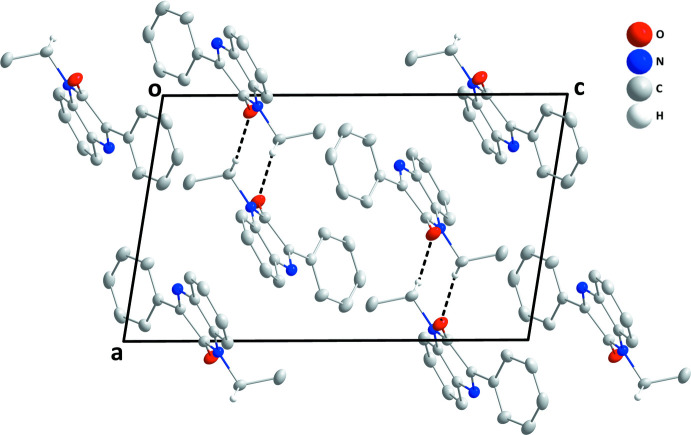
Packing view along the *b*-axis direction with C—H⋯O hydrogen bonds shown as dashed lines.

**Table 1:** Hydrogen-bond geometry (Å, °)

*D*—H⋯*A*	*D*—H	H⋯*A*	*D*⋯*A*	*D*—H⋯*A*
C9—H9*B*⋯O1^i^	0.975 (13)	2.396 (13)	3.3340 (11)	161.3 (10)

Symmetry code: (i) 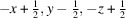.

**Table 2:** Comparison of selected (X-ray and DFT) bond lengths and angles (Å, °)

	X-ray	B3LYP/6–311G(d,p)
C1—C6	1.4071 (12)	1.4149
N2—C1	1.3846 (11)	1.3724
N2—C8	1.2983 (11)	1.299
C8—C11	1.4864 (11)	1.486
C7—C8	1.4872 (11)	1.4949
O1—C7	1.2299 (10)	1.2235
N1—C7	1.3791 (10)	1.3974
N1—C9	1.4732 (11)	1.4745
C9—C10	1.5156 (14)	1.5289
N1—C6	1.3936 (10)	1.3893
C6—N1—C9	120.63 (7)	121.2759
C7—N1—C6	122.16 (7)	122.6246
C7—N1—C9	117.14 (7)	116.0858
C8—N2—C1	119.29 (7)	120.9715
O1—C7—N1	121.54 (8)	120.1959
O1—C7—C8	123.36 (7)	124.593
N1—C9—C10	111.67 (7)	112.8427
N1—C6—C5	122.78 (8)	123.4659
N2—C8—C11	117.24 (7)	117.5205

**Table 3:** Calculated energies

Mol­ecular property	Compound (I)
Total energy *TE* (eV)	−21853.0851
*E*_HOMO_ (eV)	−6.1381
*E*_LUMO_ (eV)	−2.2463
Gap, *ΔE* (eV)	3.8918
Dipole moment, *μ* (Debye)	3.0212
Ionization potential, *I* (eV)	6.1381
Electron affinity, *A*	2.2463
Electronegativity, *χ*	4.1922
Hardness, *η*	1.9459
Electrophilicity, index *ω*	4.5158
Softness, *σ*	0.5139
Fraction of electrons transferred, *ΔN*	0.7215

**Table 4:** Experimental details

Crystal data	
Chemical formula	C_16_H_14_N_2_O
*M*_r_	250.29
Crystal system, space group	Monoclinic, *P*2_1_/*n*
Temperature (K)	150
*a*, *b*, *c* (Å)	9.2572 (9), 9.0531 (9), 15.0557 (14)
β (°)	99.329 (1)
*V* (Å^3^)	1245.1 (2)
*Z*	4
Radiation type	Mo *K*α
μ (mm^−1^)	0.09
Crystal size (mm)	0.50 × 0.47 × 0.16

Data collection	
Diffractometer	Bruker *SMART* *APEX* CCD
Absorption correction	Multi-scan (*SADABS*; Krause *et al.*, 2015 ▸)
*T*_min_, *T*_max_	0.96, 0.99
No. of measured, independent and observed [*I* > 2σ(*I*)] reflections	23365, 3364, 2925
*R*_int_	0.025
(sin θ/λ)_max_ (Å^−1^)	0.688

Refinement	
*R*[*F* ^2^ > 2σ(*F* ^2^)], *wR*(*F* ^2^), *S*	0.042, 0.130, 1.09
No. of reflections	3364
No. of parameters	228
H-atom treatment	All H-atom parameters refined
Δρ_max_, Δρ_min_ (e Å^−3^)	0.41, −0.21

Computer programs: *APEX3* and *SAINT* (Bruker, 2016 ▸), *SHELXT* (Sheldrick, 2015*a* ▸), *SHELXL2018/1* (Sheldrick, 2015*b* ▸), *DIAMOND* (Brandenburg & Putz, 2012 ▸) and *SHELXTL* (Sheldrick, 2008 ▸).

## supplementary crystallographic information

### Crystal data

**Table:**

C_16_H_14_N_2_O	*F*(000) = 528
*M**_r_* = 250.29	*D*_x_ = 1.335 Mg m^−^^3^
Monoclinic, *P*2_1_/*n*	Mo *K*α radiation, λ = 0.71073 Å
*a* = 9.2572 (9) Å	Cell parameters from 9889 reflections
*b* = 9.0531 (9) Å	θ = 2.4–29.2°
*c* = 15.0557 (14) Å	µ = 0.09 mm^−^^1^
β = 99.329 (1)°	*T* = 150 K
*V* = 1245.1 (2) Å^3^	Plate, colourless
*Z* = 4	0.50 × 0.47 × 0.16 mm

### Data collection

**Table:**

Bruker SMART APEX CCD diffractometer	3364 independent reflections
Radiation source: fine-focus sealed tube	2925 reflections with *I* > 2σ(*I*)
Graphite monochromator	*R*_int_ = 0.025
Detector resolution: 8.3333 pixels mm^-1^	θ_max_ = 29.3°, θ_min_ = 2.4°
φ and ω scans	*h* = −12→12
Absorption correction: multi-scan (*SADABS*; Krause *et al.*, 2015)	*k* = −12→12
*T*_min_ = 0.96, *T*_max_ = 0.99	*l* = −20→20
23365 measured reflections	

### Refinement

**Table:**

Refinement on *F*^2^	Primary atom site location: difference Fourier map
Least-squares matrix: full	Secondary atom site location: difference Fourier map
*R*[*F*^2^ > 2σ(*F*^2^)] = 0.042	Hydrogen site location: difference Fourier map
*wR*(*F*^2^) = 0.130	All H-atom parameters refined
*S* = 1.09	*w* = 1/[σ^2^(*F*_o_^2^) + (0.0911*P*)^2^ + 0.1012*P*] where *P* = (*F*_o_^2^ + 2*F*_c_^2^)/3
3364 reflections	(Δ/σ)_max_ < 0.001
228 parameters	Δρ_max_ = 0.41 e Å^−^^3^
0 restraints	Δρ_min_ = −0.21 e Å^−^^3^

### Special details

**Table:**

Experimental. The diffraction data were obtained from 3 sets of 400 frames, each of width 0.5° in ω, collected at φ = 0.00, 90.00 and 180.00° and 2 sets of 800 frames, each of width 0.45° in φ, collected at ω = –30.00 and 210.00°. The scan time was 10 sec/frame.
Geometry. All esds (except the esd in the dihedral angle between two l.s. planes) are estimated using the full covariance matrix. The cell esds are taken into account individually in the estimation of esds in distances, angles and torsion angles; correlations between esds in cell parameters are only used when they are defined by crystal symmetry. An approximate (isotropic) treatment of cell esds is used for estimating esds involving l.s. planes.
Refinement. Refinement of F^2^ against ALL reflections. The weighted R-factor wR and goodness of fit S are based on F^2^, conventional R-factors R are based on F, with F set to zero for negative F^2^. The threshold expression of F^2^ > 2sigma(F^2^) is used only for calculating R-factors(gt) etc. and is not relevant to the choice of reflections for refinement. R-factors based on F^2^ are statistically about twice as large as those based on F, and R- factors based on ALL data will be even larger.

### Fractional atomic coordinates and isotropic or equivalent isotropic displacement parameters (Å^2^)

**Table:**

	*x*	*y*	*z*	*U*_iso_*/*U*_eq_	
O1	0.43347 (7)	0.55741 (7)	0.27640 (5)	0.02811 (19)	
N1	0.45495 (8)	0.30830 (7)	0.26163 (5)	0.01831 (17)	
N2	0.71192 (8)	0.31976 (7)	0.38726 (5)	0.01948 (18)	
C1	0.66994 (9)	0.19050 (9)	0.34103 (6)	0.01852 (19)	
C2	0.75776 (10)	0.06426 (9)	0.36083 (6)	0.0229 (2)	
H2	0.8512 (14)	0.0771 (12)	0.4013 (9)	0.030 (3)*	
C3	0.71308 (11)	−0.07042 (10)	0.32364 (6)	0.0254 (2)	
H3	0.7731 (13)	−0.1609 (13)	0.3384 (9)	0.029 (3)*	
C4	0.57963 (11)	−0.08094 (10)	0.26512 (7)	0.0262 (2)	
H4	0.5463 (15)	−0.1782 (14)	0.2407 (10)	0.040 (4)*	
C5	0.49306 (10)	0.04224 (10)	0.24245 (6)	0.0237 (2)	
H5	0.3962 (14)	0.0325 (13)	0.2015 (9)	0.032 (3)*	
C6	0.53750 (9)	0.18006 (9)	0.28052 (6)	0.01830 (19)	
C7	0.49871 (9)	0.44227 (9)	0.30059 (6)	0.01918 (19)	
C8	0.62953 (9)	0.43608 (9)	0.37231 (5)	0.01762 (19)	
C9	0.31974 (9)	0.30696 (10)	0.19483 (6)	0.0233 (2)	
H9A	0.2631 (14)	0.3931 (15)	0.2067 (9)	0.033 (3)*	
H9B	0.2651 (13)	0.2186 (14)	0.2059 (8)	0.029 (3)*	
C10	0.35231 (12)	0.31217 (12)	0.09942 (7)	0.0316 (2)	
H10A	0.4106 (16)	0.4010 (16)	0.0903 (10)	0.043 (4)*	
H10B	0.4032 (16)	0.2247 (17)	0.0845 (10)	0.047 (4)*	
H10C	0.2620 (16)	0.3135 (15)	0.0563 (11)	0.048 (4)*	
C11	0.66935 (9)	0.56627 (9)	0.43138 (6)	0.01939 (19)	
C12	0.56472 (10)	0.66442 (10)	0.45417 (6)	0.0239 (2)	
H12	0.4642 (14)	0.6541 (13)	0.4277 (9)	0.029 (3)*	
C13	0.60553 (11)	0.77375 (11)	0.51836 (7)	0.0288 (2)	
H13	0.5343 (14)	0.8431 (14)	0.5340 (9)	0.034 (3)*	
C14	0.75007 (12)	0.78690 (11)	0.55996 (7)	0.0311 (2)	
H14	0.7760 (14)	0.8637 (16)	0.6076 (10)	0.040 (3)*	
C15	0.85551 (12)	0.69263 (11)	0.53570 (7)	0.0313 (2)	
H15	0.9599 (15)	0.7034 (14)	0.5636 (10)	0.041 (4)*	
C16	0.81542 (10)	0.58303 (10)	0.47181 (7)	0.0258 (2)	
H16	0.8876 (14)	0.5150 (15)	0.4564 (8)	0.034 (3)*	

### Atomic displacement parameters (Å^2^)

**Table:**

	*U*^11^	*U*^22^	*U*^33^	*U*^12^	*U*^13^	*U*^23^
O1	0.0285 (3)	0.0206 (3)	0.0317 (4)	0.0062 (2)	−0.0059 (3)	−0.0015 (3)
N1	0.0178 (3)	0.0182 (4)	0.0182 (3)	−0.0006 (2)	0.0006 (3)	−0.0008 (2)
N2	0.0208 (3)	0.0179 (3)	0.0192 (4)	0.0000 (2)	0.0017 (3)	0.0004 (2)
C1	0.0208 (4)	0.0172 (4)	0.0180 (4)	0.0004 (3)	0.0042 (3)	0.0004 (3)
C2	0.0256 (4)	0.0208 (4)	0.0223 (4)	0.0040 (3)	0.0043 (3)	0.0021 (3)
C3	0.0346 (5)	0.0181 (4)	0.0254 (5)	0.0048 (3)	0.0103 (4)	0.0023 (3)
C4	0.0361 (5)	0.0177 (4)	0.0264 (5)	−0.0035 (3)	0.0097 (4)	−0.0020 (3)
C5	0.0279 (4)	0.0204 (4)	0.0226 (4)	−0.0041 (3)	0.0039 (3)	−0.0012 (3)
C6	0.0215 (4)	0.0169 (4)	0.0172 (4)	−0.0006 (3)	0.0051 (3)	0.0008 (3)
C7	0.0192 (4)	0.0178 (4)	0.0200 (4)	0.0001 (3)	0.0015 (3)	−0.0010 (3)
C8	0.0182 (4)	0.0172 (4)	0.0170 (4)	−0.0010 (3)	0.0014 (3)	0.0000 (3)
C9	0.0190 (4)	0.0247 (4)	0.0244 (4)	−0.0025 (3)	−0.0021 (3)	−0.0022 (3)
C10	0.0377 (5)	0.0328 (5)	0.0216 (5)	−0.0009 (4)	−0.0036 (4)	−0.0013 (4)
C11	0.0234 (4)	0.0164 (4)	0.0178 (4)	−0.0024 (3)	0.0018 (3)	0.0005 (3)
C12	0.0258 (4)	0.0226 (4)	0.0242 (4)	−0.0026 (3)	0.0066 (3)	−0.0014 (3)
C13	0.0387 (5)	0.0233 (4)	0.0271 (5)	−0.0036 (4)	0.0137 (4)	−0.0047 (4)
C14	0.0465 (6)	0.0249 (5)	0.0217 (5)	−0.0114 (4)	0.0054 (4)	−0.0050 (4)
C15	0.0337 (5)	0.0284 (5)	0.0284 (5)	−0.0075 (4)	−0.0052 (4)	−0.0023 (4)
C16	0.0251 (4)	0.0227 (4)	0.0272 (5)	−0.0014 (3)	−0.0023 (3)	−0.0015 (3)

### Geometric parameters (Å, º)

**Table:**

O1—C7	1.2299 (10)	C9—C10	1.5156 (14)
N1—C7	1.3791 (10)	C9—H9A	0.972 (13)
N1—C6	1.3936 (10)	C9—H9B	0.975 (13)
N1—C9	1.4732 (11)	C10—H10A	0.990 (14)
N2—C8	1.2983 (11)	C10—H10B	0.966 (15)
N2—C1	1.3846 (11)	C10—H10C	0.972 (16)
C1—C2	1.4063 (11)	C11—C12	1.3976 (12)
C1—C6	1.4071 (12)	C11—C16	1.3983 (12)
C2—C3	1.3770 (13)	C12—C13	1.3920 (13)
C2—H2	0.980 (13)	C12—H12	0.955 (12)
C3—C4	1.3995 (14)	C13—C14	1.3875 (15)
C3—H3	0.995 (12)	C13—H13	0.967 (12)
C4—C5	1.3834 (13)	C14—C15	1.3893 (16)
C4—H4	0.985 (14)	C14—H14	1.000 (15)
C5—C6	1.4065 (11)	C15—C16	1.3899 (13)
C5—H5	1.006 (12)	C15—H15	0.994 (14)
C7—C8	1.4872 (11)	C16—H16	0.964 (13)
C8—C11	1.4864 (11)		
			
C7—N1—C6	122.16 (7)	N1—C9—H9A	107.0 (8)
C7—N1—C9	117.14 (7)	C10—C9—H9A	110.2 (8)
C6—N1—C9	120.63 (7)	N1—C9—H9B	107.3 (7)
C8—N2—C1	119.29 (7)	C10—C9—H9B	112.1 (7)
N2—C1—C2	118.35 (8)	H9A—C9—H9B	108.4 (11)
N2—C1—C6	121.76 (7)	C9—C10—H10A	110.7 (9)
C2—C1—C6	119.72 (8)	C9—C10—H10B	112.0 (9)
C3—C2—C1	120.54 (9)	H10A—C10—H10B	109.7 (12)
C3—C2—H2	122.3 (7)	C9—C10—H10C	110.6 (9)
C1—C2—H2	117.2 (7)	H10A—C10—H10C	109.0 (12)
C2—C3—C4	119.51 (8)	H10B—C10—H10C	104.7 (12)
C2—C3—H3	121.2 (7)	C12—C11—C16	118.94 (8)
C4—C3—H3	119.3 (7)	C12—C11—C8	122.45 (8)
C5—C4—C3	121.16 (8)	C16—C11—C8	118.36 (8)
C5—C4—H4	119.7 (8)	C13—C12—C11	120.11 (9)
C3—C4—H4	119.2 (8)	C13—C12—H12	119.7 (7)
C4—C5—C6	119.67 (9)	C11—C12—H12	120.1 (7)
C4—C5—H5	120.2 (7)	C14—C13—C12	120.51 (9)
C6—C5—H5	120.0 (7)	C14—C13—H13	118.7 (8)
N1—C6—C5	122.78 (8)	C12—C13—H13	120.8 (8)
N1—C6—C1	117.87 (7)	C13—C14—C15	119.73 (9)
C5—C6—C1	119.34 (7)	C13—C14—H14	119.0 (8)
O1—C7—N1	121.54 (8)	C15—C14—H14	121.2 (8)
O1—C7—C8	123.36 (7)	C14—C15—C16	120.02 (9)
N1—C7—C8	115.10 (7)	C14—C15—H15	120.3 (8)
N2—C8—C11	117.24 (7)	C16—C15—H15	119.7 (8)
N2—C8—C7	122.87 (7)	C15—C16—C11	120.64 (9)
C11—C8—C7	119.89 (7)	C15—C16—H16	120.3 (8)
N1—C9—C10	111.67 (7)	C11—C16—H16	119.0 (8)
			
C8—N2—C1—C2	−177.53 (8)	C1—N2—C8—C11	172.67 (7)
C8—N2—C1—C6	−2.33 (12)	C1—N2—C8—C7	−6.49 (12)
N2—C1—C2—C3	173.30 (8)	O1—C7—C8—N2	−168.24 (9)
C6—C1—C2—C3	−2.01 (13)	N1—C7—C8—N2	11.27 (12)
C1—C2—C3—C4	0.51 (14)	O1—C7—C8—C11	12.63 (13)
C2—C3—C4—C5	1.36 (14)	N1—C7—C8—C11	−167.87 (7)
C3—C4—C5—C6	−1.71 (14)	C7—N1—C9—C10	98.17 (9)
C7—N1—C6—C5	178.92 (8)	C6—N1—C9—C10	−78.78 (10)
C9—N1—C6—C5	−4.29 (13)	N2—C8—C11—C12	−148.33 (9)
C7—N1—C6—C1	−0.46 (12)	C7—C8—C11—C12	30.85 (12)
C9—N1—C6—C1	176.34 (7)	N2—C8—C11—C16	25.81 (12)
C4—C5—C6—N1	−179.18 (8)	C7—C8—C11—C16	−155.00 (8)
C4—C5—C6—C1	0.19 (13)	C16—C11—C12—C13	−2.08 (14)
N2—C1—C6—N1	5.90 (12)	C8—C11—C12—C13	172.04 (8)
C2—C1—C6—N1	−178.96 (8)	C11—C12—C13—C14	0.30 (14)
N2—C1—C6—C5	−173.50 (8)	C12—C13—C14—C15	1.64 (15)
C2—C1—C6—C5	1.64 (12)	C13—C14—C15—C16	−1.78 (15)
C6—N1—C7—O1	172.14 (8)	C14—C15—C16—C11	−0.03 (15)
C9—N1—C7—O1	−4.76 (13)	C12—C11—C16—C15	1.95 (14)
C6—N1—C7—C8	−7.37 (12)	C8—C11—C16—C15	−172.41 (9)
C9—N1—C7—C8	175.73 (7)		

### Hydrogen-bond geometry (Å, º)

**Table:**

*D*—H···*A*	*D*—H	H···*A*	*D*···*A*	*D*—H···*A*
C9—H9*B*···O1^i^	0.975 (13)	2.396 (13)	3.3340 (11)	161.3 (10)

Symmetry code: (i) −*x*+1/2, *y*−1/2, −*z*+1/2.

## References

[bb1] Abad, N., El Bakri, Y., Sebhaoui, J., Ramli, Y., Essassi, E. M. & Mague, J. T. (2018*a*). *IUCrData*, **3**, x180610.

[bb2] Abad, N., Hajji, M., Ramli, Y., Belkhiria, M., Moftah, H., Elmgirhi, S. A., Habib, M., Guerfel, T. T., Mague, J. T. & Essassi, E. M. (2020). *J. Phys. Org. Chem.* **33**, e4055.

[bb3] Abad, N., Ramli, Y., Hökelek, T., Sebbar, N. K., Mague, J. T. & Essassi, E. M. (2018*b*). *Acta Cryst.* E**74**, 1815–1820. 10.1107/S205698901801589X PMC6281099 30574380

[bb4] Becke, A. D. (1993). *J. Chem. Phys.* **98**, 5648–5652.

[bb5] Benzeid, H., Bouhfid, R., Massip, S., Leger, J. M. & Essassi, E. M. (2011). *Acta Cryst.* E**67**, o2990. 10.1107/S1600536811042474 PMC3247392 22220010

[bb6] Brandenburg, K. & Putz, H. (2012). *DIAMOND*, Crystal Impact GbR, Bonn, Germany.

[bb7] Bruker (2016). *APEX3*, *SADABS* and *SAINT*. Bruker AXS Inc., Madison, Wisconsin, USA.

[bb8] Chkirate, K., Fettach, S., El Hafi, M., Karrouchi, K., Elotmani, B., Mague, J. T., Radi, S., Faouzi, M. E. A., Adarsh, N. N., Essassi, E. M. & Garcia, Y. (2020*a*). *J. Inorg. Biochem.* **208**, 111092. 10.1016/j.jinorgbio.2020.111092 32461023

[bb9] Chkirate, K., Fettach, S., Karrouchi, K., Sebbar, N. K., Essassi, E. M., Mague, J. T., Radi, S., Faouzi, M. E. A., Adarsh, N. N. & Garcia, Y. (2019). *J. Inorg. Biochem.* **191**, 21–28. 10.1016/j.jinorgbio.2018.11.006 30448715

[bb10] Chkirate, K., Karrouchi, K., Dege, N., Sebbar, N. K., Ejjoummany, A., Radi, S., Adarsh, N. N., Talbaoui, A., Ferbinteanu, M., Essassi, E. M. & Garcia, Y. (2020*b*). *New J. Chem.* **44**, 2210–2221.

[bb11] Daouda, B., Doumbia, M. L., Essassi, E. M., Saadi, M. & El Ammari, L. (2013). *Acta Cryst.* E**69**, o662. 10.1107/S1600536813008702 PMC3647858 23723824

[bb12] Frisch, M. J., Trucks, G. W., Schlegel, H. B., Scuseria, G. E., Robb, M. A., Cheeseman, J. R., Scalmani, G., Barone, V., Mennucci, B., Petersson, G. A., Nakatsuji, H., Caricato, M., Li, X., Hratchian, H. P., Izmaylov, A. F., Bloino, J., Zheng, G., Sonnenberg, J. L., Hada, M., Ehara, M., Toyota, K., Fukuda, R., Hasegawa, J., Ishida, M., Nakajima, T., Honda, Y., Kitao, O., Nakai, H., Vreven, T., Montgomery, J. A. Jr, Peralta, J. E., Ogliaro, F., Bearpark, M., Heyd, J. J., Brothers, E., Kudin, K. N., Staroverov, V. N., Kobayashi, R., Normand, J., Raghavachari, K., Rendell, A., Burant, J. C., Iyengar, S. S., Tomasi, J., Cossi, M., Rega, N., Millam, J. M., Klene, M., Knox, J. E., Cross, J. B., Bakken, V., Adamo, C., Jaramillo, J., Gomperts, R., Stratmann, R. E., Yazyev, O., Austin, A. J., Cammi, R., Pomelli, C., Ochterski, J. W., Martin, R. L., Morokuma, K., Zakrzewski, V. G., Voth, G. A., Salvador, P., Dannenberg, J. J., Dapprich, S., Daniels, A. D., Farkas, O., Foresman, J. B., Ortiz, J. V., Cioslowski, J. & Fox, D. J. (2009). *GAUSSIAN09.* Rev. A.02. Gaussian Inc., Wallingford, CT, USA.

[bb13] Galal, S. A., Khairat, S. H. M., Ragab, F. A. F., Abdelsamie, A. S., Ali, M. M., Soliman, S. M., Mortier, J., Wolber, G. & El Diwani, H. I. (2014). *Eur. J. Med. Chem.* **86**, 122–132. 10.1016/j.ejmech.2014.08.048 25147154

[bb14] Groom, C. R., Bruno, I. J., Lightfoot, M. P. & Ward, S. C. (2016). *Acta Cryst.* B**72**, 171–179. 10.1107/S2052520616003954 PMC4822653 27048719

[bb15] Hirshfeld, H. L. (1977). *Theor. Chim. Acta*, **44**, 129–138.

[bb16] Holschbach, M. H., Bier, D., Wutz, W., Sihver, W., Schüller, M. & Olsson, R. A. (2005). *Eur. J. Med. Chem.* **40**, 421–437. 10.1016/j.ejmech.2004.12.005 15893016

[bb17] Krause, L., Herbst-Irmer, R., Sheldrick, G. M. & Stalke, D. (2015). *J. Appl. Cryst.* **48**, 3–10. 10.1107/S1600576714022985 PMC4453166 26089746

[bb18] Mamedov, V. A., Kalinin, A. A., Gubaidullin, A. T., Isaikina, O. G. & Litvinov, I. A. (2005*a*). *Zh. Org. Khim.* **41**, 609–616.

[bb19] Mamedov, V. A., Kalinin, A. A., Yanilkin, V. V., Gubaidullin, A. T., Latypov, Sh. K., Balandina, A. A., Isaikina, O. G., Toropchina, A. V., Nastapova, N. V., Iglamova, N. A. & Litvinov, I. A. (2005*b*). *Izv. Akad. Nauk, Ser. Khim.* **11**, 2534–2542.

[bb20] McKinnon, J. J., Jayatilaka, D. & Spackman, M. A. (2007). *Chem. Commun.* pp. 3814. 10.1039/b704980c 18217656

[bb21] Ramli, Y., El Bakri, Y., El Ghayati, L., Essassi, E. M. & Mague, J. T. (2018). *IUCrData*, **3**, x180390.

[bb22] Sheldrick, G. M. (2008). *Acta Cryst.* A**64**, 112–122. 10.1107/S0108767307043930 18156677

[bb23] Sheldrick, G. M. (2015*a*). *Acta Cryst.* A**71**, 3–8.

[bb24] Sheldrick, G. M. (2015*b*). *Acta Cryst.* C**71**, 3–8.

[bb25] Sun, L.-R., Li, X., Cheng, Y.-N., Yuan, H.-Y., Chen, M.-H., Tang, W., Ward, S. G. & Qu, X.-J. (2009). *Biomed. Pharmacother.* **63**, 202–208. 10.1016/j.biopha.2008.07.090 18818047

[bb26] Turner, M. J., McKinnon, J. J., Wolff, S. K., Grimwood, D. J., Spackman, P. R., Jayatilaka, D. & Spackman, M. A. (2017). *CrystalExplorer17* *The University of Western Australia*.

